# Quasi-Static Variation of Power-Law and Log-Normal Distributions of Urban Population

**DOI:** 10.3390/e23070908

**Published:** 2021-07-17

**Authors:** Atushi Ishikawa, Shouji Fujimoto, Arturo Ramos, Takayuki Mizuno

**Affiliations:** 1 Department of Economic Informatics, Kanazawa Gakuin University, Kanazawa 920-1392, Japan; fujimoto@kanazawa-gu.ac.jp; 2 Department of Economic Analysis, Universidad de Zaragoza, 50005 Zaragoza, Spain; aramos@unizar.es; 3 National Institute of Informatics, Tokyo 101-8430, Japan; mizuno@nii.ac.jp; 4 The Graduate University for Advanced Studies [SOKENDAI], Kanagawa 240-0193, Japan; 5 Center for Advanced Research in Finance, The University of Tokyo, Tokyo 113-0033, Japan

**Keywords:** urban population, power law, log-normal distribution, Gibrat’s law, non-gibrat property, quasi-time-reversal symmetry

## Abstract

We analytically derived and confirmed by empirical data the following three relations from the quasi-time-reversal symmetry, Gibrat’s law, and the non-Gibrat’s property observed in the urban population data of France. The first is the relation between the time variation of the power law and the quasi-time-reversal symmetry in the large-scale range of a system that changes quasi-statically. The second is the relation between the time variation of the log-normal distribution and the quasi-time-reversal symmetry in the mid-scale range. The third is the relation among the parameters of log-normal distribution, non-Gibrat’s property, and quasi-time-reversal symmetry.

## 1. Introduction

In natural science, power-law distributions of various physical quantities are observed in phase transitions and critical phenomena [1]. In social sciences, power-law distributions are obtained in various observables, such as urban population, personal income, sales, assets, and the number of employees which represent the size of firms (firm size variables) [2]. Here, the power-law exponent of firm size variables reportedly changes little with time [3]. However, it has not been confirmed whether distribution is actually stable over time in the event of such huge economic upheavals as World War II, the Great Depression, and the Covid-19 pandemic because the long-term, historical financial data of firms are difficult to obtain and cannot be directly observed.

In response to this problem, we observed a temporal change in the power-law distributions in the large-scale range of land values in Japan [4,5]. We analytically derived the time-varying, power-law distribution from Gibrat’s law and the quasi-time-reversal symmetry observed in the system of land values. We derived the analytical relationship between the inclination of the symmetry axis of quasi-time inversion and the time change of the power-law exponent and observed it accurately in empirical data. Here, Gibrat’s law is a property where the growth-rate distribution of the observables (in the present case, published land prices) does not depend on the initial value [6,7]. We also describe a quasi-inversion symmetry between different types of firm size variables at the same time [8] and identify it with an economically important Cobb-Douglas type production function [9]. However, it remains unclear whether there is any consistency between quasi-time-reversal symmetry and the time change of the power-law exponent in other data in which the long-term time changes of macroscopic systems are observed.

Therefore, we focused on urban population as data because they are publicly disclosed, long-term data that are relatively accessible whose distribution may have changed significantly over time, similar to published land prices in Japan. In our previous study [10], we superimposed data of urban population from eleven census surveys over 110 years in the United States, Italy, and Spain (period 10) and confirmed that the distribution of growth rates was regularly dependent on the initial values. We call this initial dependence non-Gibrat’s property. We showed by numerical simulation that the long-term growth of urban population, which is confirmed in empirical data, can be explained by the non-Gibrat’s property. In this analysis, the amount of census data on urban populations was approximately 10,000 per period for each of the three countries, (the United States, Italy, and Spain), so we superimposed the data to enhance the statistics and capture the macroscopic nature of the census. No temporal change was observed in the distribution of the urban population and its growth rate.

This paper newly examines 140 years’ worth of urban population data (30 periods) in France provided by the French National Institute of Statistical and Economic Research [11]. The database contains around 35,000 urban population data per term, per commune, which is the country’s smallest administrative division. Such an amount of data makes it possible to observe changes in urban population distribution and its growth-rate distribution over time without the need to overlay the data to capture statistical macroscopic properties, as in our previous study [10]. Therefore, we can confirm the consistency of the time change of quasi-time-reversal symmetry and power-law distribution which could not be tackled in our previous study. Furthermore, we can confirm not only the temporal change of the power-law distribution in the large-scale range but also the consistency between the temporal changes of the log-normal distribution in the mid-scale range and the quasi-time-reversal symmetry. In addition, we discussed the consistency of the parameters of log-normal distribution and non-Gibrat’s property of the mid-scale range in a system in which the distributions change quasi-statically, which was not captured even in the analysis of land price data [4,5].

The structure of this paper is as follows. Section 2 describes the urban population data for France. Then, power-law and log-normal distributions, which were observed in the large- and mid-scale ranges of the data and their time variations, are discussed analytically. Quasi-time-reversal symmetry, large-scale Gibrat’s law, and mid-scale non-Gibrat’s property are important concepts in this discussion. In Section 3, we first confirm the analytical results in Section 2 by observing the relationship between the slope of the symmetry axis of quasi-time inversion and the time variation of the power-law exponent and the logarithmic standard deviation in the empirical data. Then, we show the consistency between the parameters of the mid-scale non-Gibrat’s property and those of log-normal distribution even in a quasi-statically changing system. Finally, Section 4 summarizes this paper and presents future issues.

## 2. Materials and Methods

### 2.1. Data

This paper analyzes the database of the National Institute of Statistical Economics in France [11], “Detailed Figures-Past Population Series (1876 to 2017)”. In France, the smallest administrative division is called a commune, and the database contains around 35,000 communes (except those of the French region of Mayotte) from 1876 to 2017 (Table 1 for details). The database includes annual data since 2006. Previously, the observation intervals of the data were inconsistent, often five year periods. In this paper, we assigned serial number *i* to 31 observation years (i=0,1,2,⋯,30) (Table 1). World War I’s observation interval was a 10-year period from 1911 to 1921, and World War II’s was 18 years from 1936 to 1954. Table 1 shows a population decrease after World War I and only a slight increase in the 18 years since the end of World War II. This paper deals with the changes in the short-term properties of two consecutive periods. For the observations in Table 1 from 1931 to 1936, 1936 to 1954, and 1954 to 1962, the number of communes changed in two successive periods. In these cases, we analyzed only communes in both years.

### 2.2. Power and Log-Normal Distributions and Their Changes

**Figure 1:**
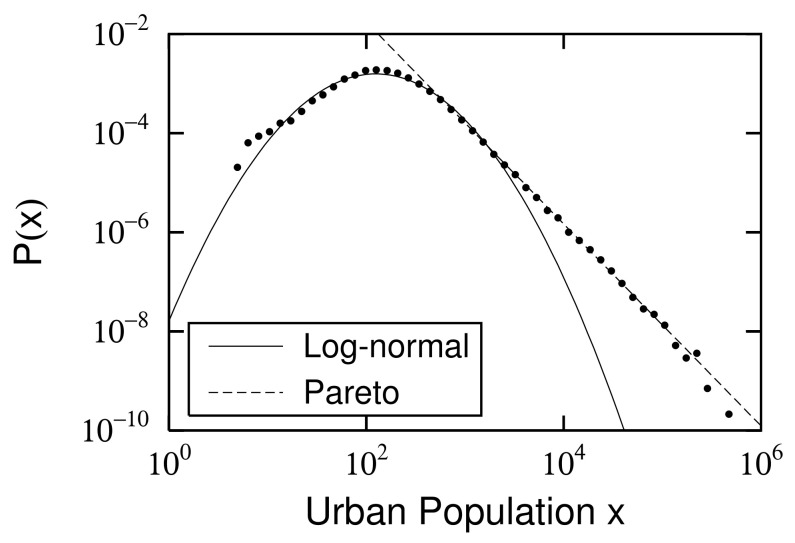
Urban population (*x*) distribution in France in 1962: Power law (1) is applied to top 10% of data, and the exponent is μ=1.05±0.00. However, we excluded top 0.1% of fluctuating data. Logarithmic standard deviation, calculated by fitting mid-scale range to log-normal distribution (2) with $x_{\min}=10$ and $x_{0}=3,100$, is σ=0.70±0.02. Here, we estimated μ and σ using least-square method.

**Figure 2:**
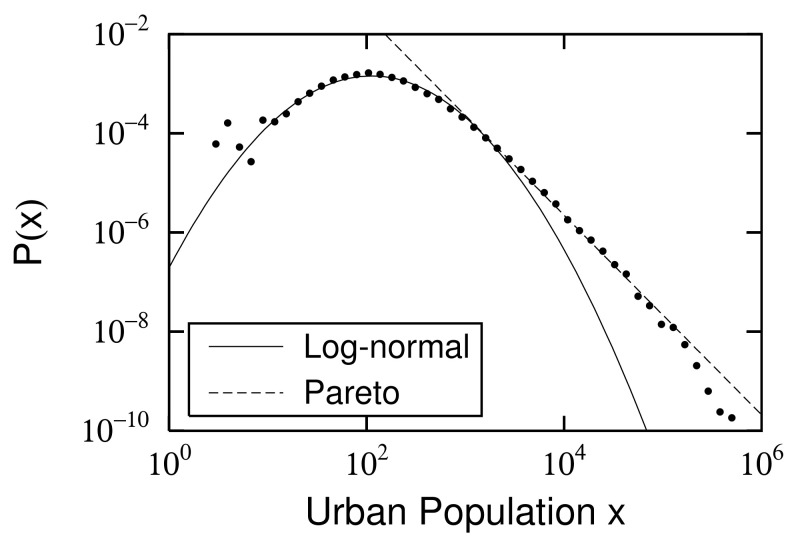
Urban population (*x*) distribution in France in 2015: Power law (1) is applied to top 10% of data, and power exponent is μ=1.15±0.00. However, we excluded the top 0.1% of fluctuating data. Logarithmic standard deviation, calculated by fitting mid-scale range to log-normal distribution (2) with $x_{\min}=10$ and $x_{0}=3,100$, is σ=0.78±0.01. Here, we estimated μ and σ using least-square method.

As typical examples, the probability density functions (PDFs) of urban population (x) in France in 1962 and 2015 are shown in Figure 1 and Figure 2. In each figure, a power-law distribution [12,13,14,15],

(1) $$\begin{array}{c} P\left(x\right)=Cx^{-\mu -1}\;\;\;\;\mathrm{for}\;\;\;\;x_{0}<x\;, \end{array}$$

is observed in the large-scale range. Here, exponent μ parameterizes the spread of the power-law distribution as 1/μ, and $x_{0}$ is the lower limit of the large-scale range. At the same time, a log-normal distribution,

(2) $$\begin{array}{c} P\left(x\right)=Cx^{-\mu -1}\;\exp\left[-\alpha \ln^{2}\frac{x}{x_{0}}\right]\;\;\;\;\;\mathrm{for}\;\;x_{\min}<x<x_{0}\;, \end{array}$$

is also observed in each mid-scale range [6,16,17]. $x_{\min}$ is the lower limit of the mid-scale range. α is a parameter that is related to logarithmic standard deviation σ of the log-normal distribution described in the standard way:

(3) $$\begin{array}{c} \alpha =\frac{1}{2\sigma^{2}}\;. \end{array}$$

**Figure 3:**
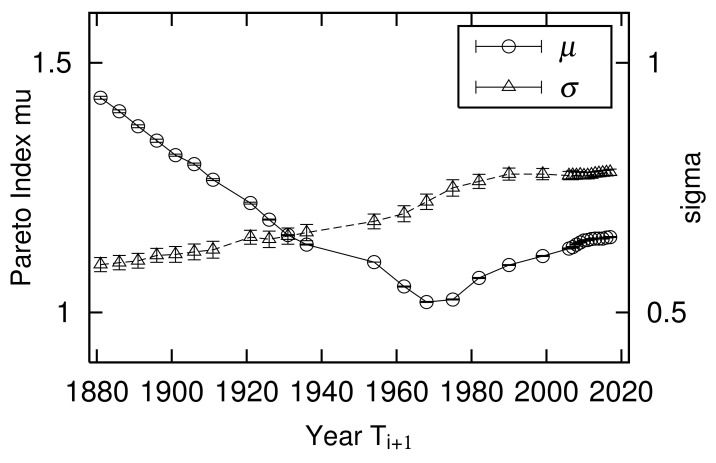
Temporal changes in power-law index μ and logarithmic standard deviation σ of urban population in France from 1881 to 2017.

Power-law exponents μ of the large-scale range and log-normal standard deviation σ of the mid-scale range are different in both years. This is not a special case; both constantly changed between 1881 and 2017 (Figure 3). Power exponent μ varies from approximately 1 to 1.5, where the smallest value is μ=1.02±0.00 in 1968. The extent of the large-scale range following the power-law distribution is characterized by 1/μ. Figure 3 shows that the spread of urban population in the large-scale range was greatest in 1968 during a period of riots, strikes, and demonstrations in Paris, which became known as “May 1968”. The French economy was experiencing high economic growth. The power-law index continued to increase as rapid economic growth ended and the population that was concentrated in large cities spread to rural areas after the strikes and riots ended. Logarithmic standard deviation σ varied from about 0.6 to 0.8, with the smallest in 1881 at σ=0.60±0.01 and the largest value in 2017 at σ=0.78±0.01. This indicates that the expansion of the mid-scale urban population distribution grew year by year, especially in the years following 1968. The population moved from large cities to rural areas, as described above. Here, we estimated μs and σs, applying the same least-square methods in Figure 1 and Figure 2.

### 2.3. Quasi-Time-Reversal and Changes

**Figure 4:**
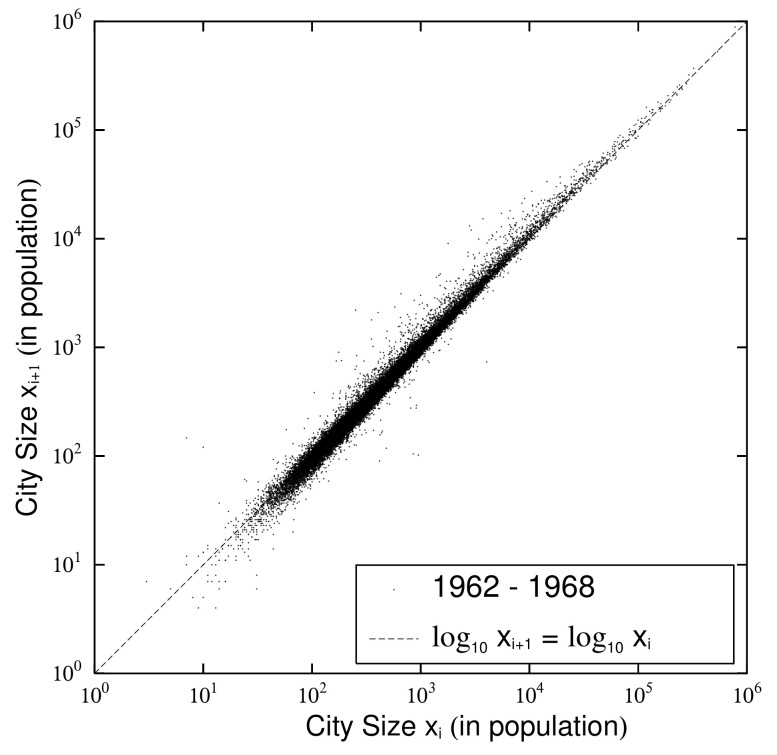
Scatter plots of urban population $\left(x_{i},x_{i+1}\right)$ for 1962 $\left(=T_{i}\right)$ and 1968 $\left(=T_{i+1}\right)$ with i=13 in Table 1.

**Figure 5:**
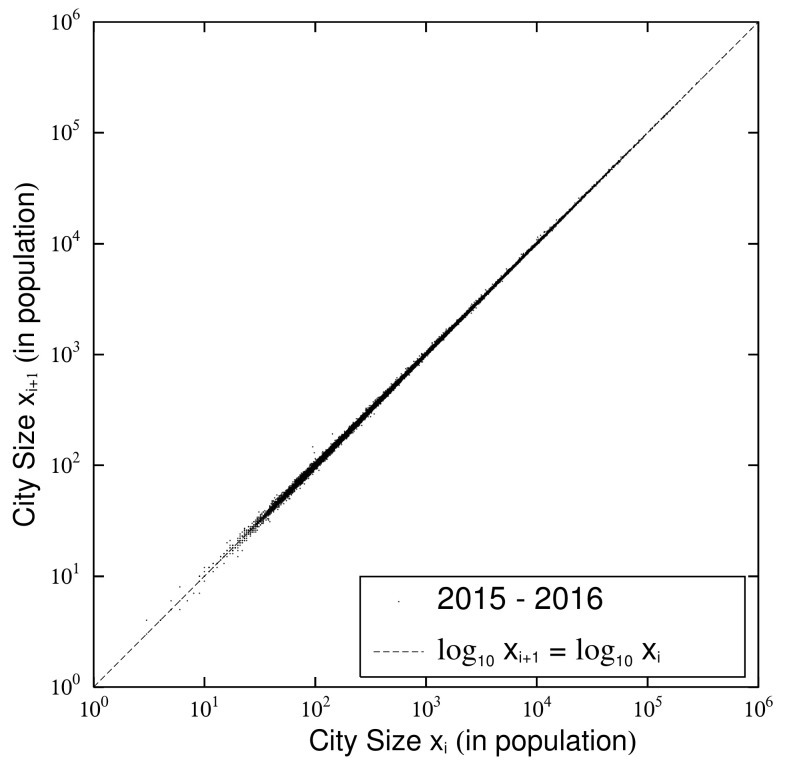
Scatter plots of urban population $\left(x_{i},x_{i+1}\right)$ for 1962 $\left(=T_{i}\right)$ and 1968 $\left(=T_{i+1}\right)$ with i=13 in Table 1.

Next, we observe the fluctuation in urban population that affected the change in the exponent and the logarithmic standard deviation in the previous subsection (Figure 3) by a scatter plot of urban population at successive measurement points. Figure 4 is a scatter plot of the urban population of $\left(x_{i}\right)$ in 1962 and $\left(x_{i+1}\right)$ in 1968 with i=13 in Table 1. Figure 5 is a scatter plot of the urban population of $\left(x_{i}\right)$ in 2015 and $\left(x_{i+1}\right)$ in 2016 with i=28 in Table 1. The dots in each figure represent cities. Figure 4 shows the changes in the urban population during the six-year period when the survey was conducted, and its population changes are larger than those in Figure 5, which shows them over 1 year. In Figure 4 and Figure 5, cities with unchanged population are on the dotted line: $\log_{10}x_{i+1}=\log_{10}x_{i}$. Cities with increasing populations are above the dotted line; cities with decreasing populations are below it. Urban populations, unlike firm size variables, rarely grow by 10 fold or 1/10 in the short term. Therefore, the urban population plotted in Figure 4 and Figure 5 is distributed symmetrically with respect to the line along which the vertical and horizontal axes are approximately equal.

If this symmetry is strictly true, the system is in equilibrium. Fujiwara et al. found that firm size variables were in such a state and called it time-reversal symmetry: $x_{i}↔x_{i+1}$ [18,19]. However, as seen in Table 1, the urban population increases in almost every period, and strictly speaking, no time-reversal symmetry was established. In our previous work [4,5], we found that posted land prices in Japan are in a similar situation. Assuming that the system’s time evolution is quasi-static, the system is symmetric with respect to the following line: $\log_{10}x_{i+1}=\theta \;\log_{10}x_{i}+\log_{10}a$. Here, θ and $\log_{10}a$ are the slope and intercept of the line (axis of symmetry).

When a system, composed of a large number of variables, changes quasi-statically, it is symmetric with respect to time inversion: $a{x_{i}}^{\theta }↔x_{i+1}$. This is a quasi-static extension of time-reversal symmetry, which we call quasi-time-reversal symmetry and express using the following joint PDF:

(4) $$\begin{array}{ccc} & & P_{J}\left(x_{i},x_{i+1}\right)\;dx_{i}\;dx_{i+1}\\ & & =P_{J}\left({\left(\frac{x_{i+1}}{a}\right)}^{1/\theta },a\;{x_{i}}^{\theta }\right)\;d\left({\left(\frac{x_{i+1}}{a}\right)}^{1/\theta }\right)\;d\left(a\;{x_{i}}^{\theta }\right). \end{array}$$

**Figure 6:**
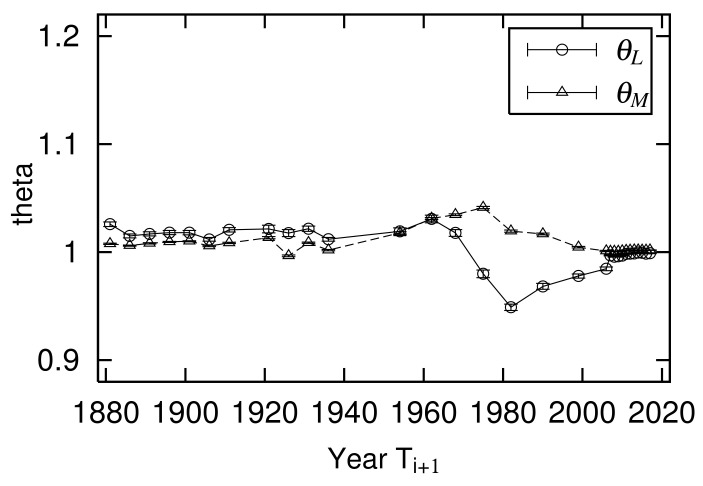
Slope of quasi-time-reversal-symmetry axis θ in large- and mid-scale ranges: Large- and mid-scale parameters $\theta_{L}$ and $\theta_{M}$ were evaluated by linear regression analysis for regions of $x_{i},x_{i+1}>x_{0}$, $x_{0}>x_{i},x_{i+1}>x_{\min}$.

Figure 6 shows a time transition in the large- and mid-scale ranges of slope θ of the axis of symmetry of the quasi-time inversion. $\theta_{L}$ is the slope of the symmetry axis in the large-scale region measured with $x_{0}<x_{i},x_{i+1}$, and $\theta_{M}$ is the slope of the symmetry axis in the mid-scale region measured with $x_{\min}<x_{i},x_{i+1}<x_{0}$. Linear regression analysis was used to measure the parameters in the large- and mid-scale regions. This step is approximately allowed when the data’s scatter plots are concentrated on the axis of symmetry, such as urban populations and land prices. Conversely, if the scatter plot has a very large variance, such as firm size variables, it is inappropriate to use regression analysis to identify the axis of symmetry because the results are different when the explanatory and objective variables are interchanged. In such cases, the axis of symmetry can be captured not by regression analysis but by the index of surface openness [20,21,22] used in geomorphology [8,23]. Figure 6 shows that the slope of quasi-time-reversal symmetry axis θ often fluctuates slightly above 1. This reflects an overall increase in urban population. As an exception, $\theta_{L}$ fell well below 1 between 1968 and 1999, reflecting that a large amount of the city population moved to mid-sized cities.

### 2.4. Gibrat’s Law and Non-Gibrat’s Property

In this paper, we discuss the time variations of the power-law and log-normal distributions in the large- and mid-scale ranges. The distribution’s temporal variation is caused by the quasi-time-reversal symmetry introduced in the previous subsection. On the other hand, the basis of the power-law and log-normal distributions is Gibrat’s law and the non-Gibrat’s property introduced in this subsection. As mentioned in the Introduction, Gibrat’s law is a property where the growth-rate distribution of the variables of a system is independent of the initial value. Gibrat’s law resembles Gibrat’s process in the multiplication stochastic process [2], except that it can be observed in empirical data. Fujiwara et al. showed analytically that by combining Gibrat’s law with time-reversal symmetry, a power law in an equilibrium state can be derived and confirmed by empirical data [18,19]. An extension to a system that changes quasi-statically is the quasi-time-reversal symmetry described in the previous subsection. The non-Gibrat’s property discussed here extends this discussion to mid-scale log-normal distribution [24,25].

**Figure 7:**
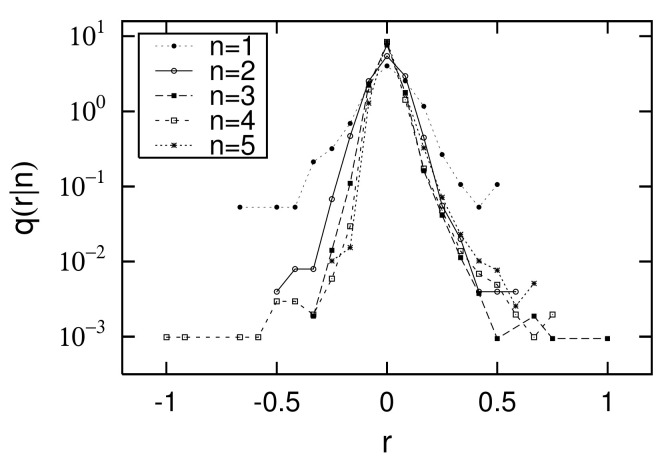
Conditional PDFs $q(r|x_{i})$ or q(r|n) of log growth rate of urban population $r=\log_{10}x_{i+1}/x_{i}$, calculated using data plotted in Figure 4 for 1962 and 1968 (i=13): Initial value $x_{i}$ is contained in five logarithmically equal-sized bins: $x_{i}\in \left[10^{1+0.5(n-1)},10^{1+0.5n}\right)$(n=1,2,⋯,5). The data range shown here is $10^{1}\leq x_{i}<10^{3.5}$ (population).

**Figure 8:**
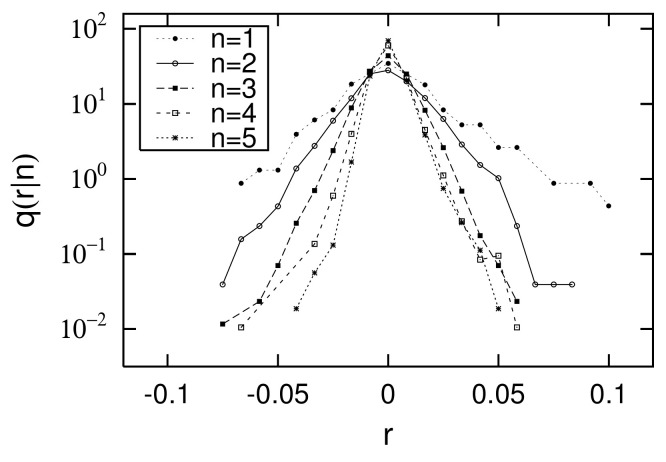
Conditional PDFs $q(r|x_{i})$ or q(r|n) of log growth rate of urban population $r=\log_{10}x_{i+1}/x_{i}$, calculated using data plotted in Figure 5 for 2015 and 2016 (i=28): Initial value $x_{i}$ is contained in five logarithmically equal-sized bins: $x_{i}\in \left[10^{1+0.5(n-1)},10^{1+0.5n}\right)$(n=1,2,⋯,5). Data range shown here is $10^{1}\leq x_{i}<10^{3.5}$ (population).

Figure 7 and Figure 8 show the distributions of the logarithmic growth rate of the urban population from 1962 to 1968 (i=13) and from 2015 to 2016 (i=28): $r=\log_{10}R=\log_{10}x_{i+1}/x_{i}$. Each figure observes conditional PDFs: $Q(R|x_{i})$, where the initial value of $x_{i}$ is placed in 5 bins: $x_{i}\in \left[10^{1+0.5(n-1)},10^{1+0.5n}\right)$(n=1,2,⋯,5). $q(r|x_{i})$, which is a PDF of logarithmic growth rate *r*, is related to $Q(R|x_{i})$ by the following relation:

(5) $$\begin{array}{c} \log_{10}q\left(r|x_{i}\right)=\log_{10}Q\left(R|x_{i}\right)+r+\log_{10}\left(\ln10\right)\;. \end{array}$$

In Figure 7 and Figure 8, we confirm that the width of the growth-rate distributions decreases in both the right and left directions as *n* increases, that is, as initial value $x_{i}$ increases. At the same time, we also confirm that as initial value $x_{i}$ increases, the dependency of the growth-rate distribution on the initial value decreases. When the initial value dependency is negligible, we call the property Gibrat’s law, which can be expressed as follows [18,19]:

(6) $$\begin{array}{c} Q\left(R|x_{i}\right)=Q\left(R\right)\;\;\;\mathrm{for}\;\;\;x_{0}<x_{i}\;. \end{array}$$

At the same time, from Figure 7 and Figure 8, we assume that the shape of the growth-rate distribution can be approximated on both logarithmic axes by the following equations with a curvature convex downward:

(7) $$\begin{array}{ccc} \log_{10}q\left(r|x_{i}\right) & = & c-t_{+}\left(x_{i}\right)\;r+\ln10\;u_{+}\left(x_{i}\right)r^{2}\;\;\;\mathrm{for}\;\;\;r>0\;, \end{array}$$

(8) $$\begin{array}{ccc} \log_{10}q\left(r|x_{i}\right) & = & c+t_{-}\left(x_{i}\right)\;r+\ln10\;u_{-}\left(x_{i}\right)r^{2}\;\;\;\mathrm{for}\;\;\;r<0\;. \end{array}$$

Here, we assume that *r* has an appropriate cut-off, $r_{c}$, and that the PDF’s integral does not diverge. However, $r_{c}$ is not explicitly expressed in Equations (7) and (8). This approximation was first proposed for the sales growth-rate distributions of firms [26,27]. In many cases, downward convex growth-rate distributions are observed on both the logarithmic axes for such non-negative observations as firms’ sales, number of employees, or each country’s GDP [28]. Figure 7 and Figure 8 differ greatly in the range of *r*. The width of the growth-rate distribution calculated from the scatter plot in Figure 4 is −1<r<1, while that in Figure 5 is −0.1<r<0.1. This is because the time interval in the former scatter plot is six years, but, in the latter, it is only one year, resulting in a difference in the growth width. In spite of this difference, the curvature of the downward convex growth-rate distribution is observed in both figures.

**Figure 9:**
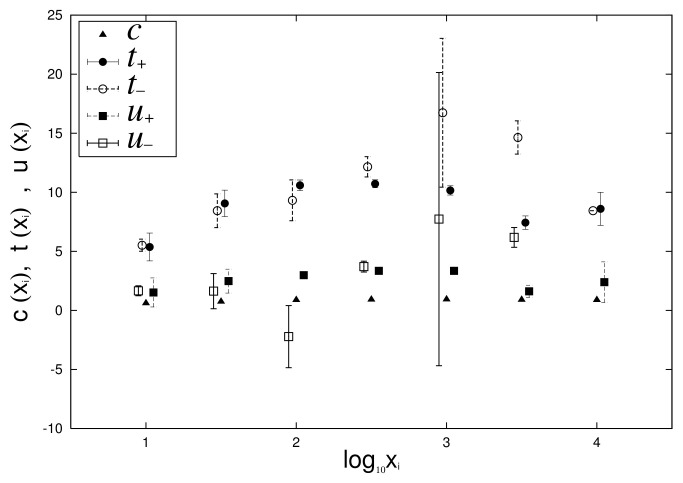
$\log_{10}x_{i}$ dependencies of $t_{\pm }$, $u_{\pm }$, and *c* are evaluated by applying Equations (7) and (8) to conditional growth-rate distributions in Figure 7 for 1962 and 1968 (i=13). To simplify graph comprehension, coordinates of horizontal axis of each point are shifted slightly.

**Figure 10:**
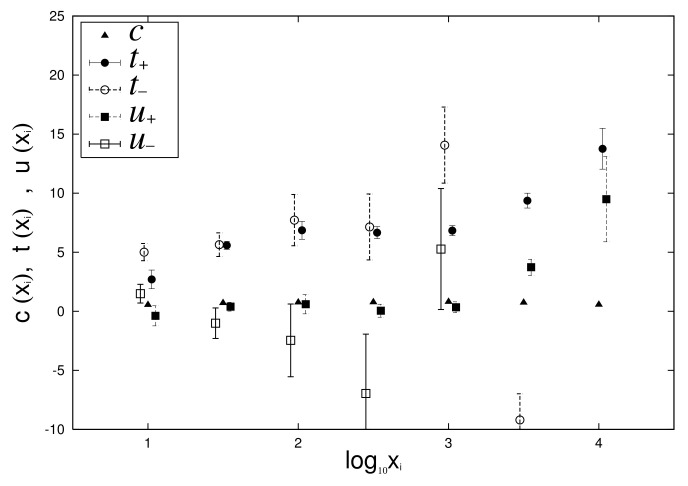
$\log_{10}x_{i}$ dependencies of $t_{\pm }$, $u_{\pm }$, and *c* are evaluated by applying Equations (7) and (8) to conditional growth-rate distributions in Figure 8 for 2015 and 2016 (i=28). To simplify graph comprehension, coordinates of horizontal axis of each point are shifted slightly.

Figure 9 and Figure 10 depict the dependency of *c*, $t_{\pm }$, and $u_{\pm }$ on $\log_{10}x_{i}$ by applying Equations (7) and (8) to Figure 7 and Figure 8. From Figure 9 and Figure 10, we confirm that the approximation that *c* has no dependency on $x_{i}$ is valid in Equations (7) and (8) of the growth-rate distribution.

### 2.5. Quasi-Static Change of Power-Law Distribution

In this subsection, we analytically show that the time variation of the power law (1) is derived from Gibrat’s law, (6), and quasi-time-reversal symmetry, (4) [4]. In the next subsection, we analytically show that non-Gibrat’s property, (7), (8), and quasi-time-reversal symmetry, (4), lead to the time variation of the log-normal distribution, (2) [5].

Quasi-time-reversal symmetry, (4), using extended growth rate $R=x_{i+1}/a{x_{i}}^{\theta }$, is rewritten by variables $x_{i},R$:

(9) $$\begin{array}{c} P_{J}\left(x_{i},R\right)dx_{i}dR=P_{J}\left(R^{1/\theta }x_{i},R^{-1}\right)d\left(R^{1/\theta }x_{i}\right)d\left(R^{-1}\right). \end{array}$$

This leads to

(10) $$\begin{array}{c} P_{J}\left(x_{i},R\right)=R^{1/\theta -2}\;P_{J}\left(R^{1/\theta }x_{i},R^{-1}\right)\;. \end{array}$$

Using conditional PDF $Q\left(R|x_{T}\right)=P_{J}\left(x_{i},R\right)/P\left(x_{i}\right)$ and Gibrat’s law, (6), this is reduced to

(11) $$\begin{array}{c} \frac{P(x_{i})}{P(R^{1/\theta }x_{i})}=R^{1/\theta -2}\frac{Q(R^{-1}|R^{1/\theta }x_{i})}{Q(R|x_{i})}=R^{1/\theta -2}\frac{Q(R^{-1})}{Q(R)}\;. \end{array}$$

Here, we assume that Gibrat’s law, (6), holds under a transformation: $x_{i}↔R^{1/\theta }x_{i}\left(={\left(x_{i+1}/a\right)}^{1/\theta }\right)$. This is valid in a system that has quasi-time-reversal symmetry. Since the last term in Equation (11) is only a function of *R*, we signify it by $G_{\theta }\left(R\right)$ and expand Equation (11) to *R* around 1 as R=1+ϵ(ϵ≪1). The 0-th order of ϵ is a trivial expression, and the 1-st order term yields the following differential equation:

(12) $$\begin{array}{c} {G_{\theta }}^{\prime }\left(1\right)P\left(x_{i}\right)+\frac{x_{i}}{\theta }\frac{d}{dx_{i}}P\left(x_{i}\right)=0\;. \end{array}$$

Here, ${G_{\theta }}^{\prime }\left(\cdot \right)$ denotes the *R* differentiation of $G_{\theta }\left(\cdot \right)$. No further useful information comes from the second and higher order terms of ϵ. The solution to this differential equation is uniquely given:

(13) $$\begin{array}{c} P\left(x_{i}\right)\propto {x_{i}}^{-\theta {G_{\theta }}^{\prime }\left(1\right)}\;. \end{array}$$

This solution satisfies Equation (11), even if *R* is not near R=1, when $Q\left(R\right)=R^{-{G_{\theta }}^{\prime }\left(1\right)-1}Q\left(R^{-1}\right)$ holds. This is called the reflection law [18,19].

Next, in quasi-static system $\left(x_{i},x_{i+1}\right)$, we identify distribution $P(x_{i+1})$. Here, $P_{x_{i}}\left(x_{i}\right)$, $P_{x_{i+1}}\left(x_{i+1}\right)$ are collectively written as $P(x_{i})$, $P(x_{i+1})$, for simplicity. From Equation (13) and $P\left(x_{i}\right)dx_{i}=P\left(x_{i+1}\right)dx_{i+1}$, $P(x_{i+1})$ can be expressed:

(14) $$\begin{array}{c} P\left(x_{i+1}\right)=P\left(x_{i}\right)\frac{dx_{i}}{dx_{i+1}}\propto {x_{i+1}}^{-{G_{\theta }}^{\prime }\left(1\right)+1/\theta -1}\;. \end{array}$$

Here, we denote power-law indices at *i*, i+1 by $\mu_{i}$, $\mu_{i+1}$ and represent $P(x_{i})$, $P(x_{i+1})$ as follows:

(15) $$\begin{array}{c} P\left(x_{i}\right)\propto {x_{i}}^{-\mu_{i}-1}\;,\;\;\;P\left(x_{i+1}\right)\propto {x_{i+1}}^{-\mu_{i+1}-1}\;. \end{array}$$

Comparing Equations (13) and (14) to Equation (15), we obtain $\theta {G_{\theta }}^{\prime }\left(1\right)=\mu_{i}+1$, ${G_{\theta }}^{\prime }\left(1\right)-1/\theta +1=\mu_{i+1}+1$ and conclude the relation among $\mu_{i}$, $\mu_{i+1}$, and θ as follows:

(16) $$\begin{array}{c} \theta =\frac{\mu_{i}}{\mu_{i+1}}=\frac{1/\mu_{i+1}}{1/\mu_{i}}\;. \end{array}$$

From this expression, we understand that the slope of the symmetry axis of time inversion θ represents the rate of the change of Pareto indices $\mu_{i}$, $\mu_{i+1}$ at *i*, i+1. This idea is geometrically consistent since the width of the power law at *i*, i+1 can be expressed as $1/\mu_{i}$, $1/\mu_{i+1}$ on the logarithmic axis.

### 2.6. Quasi-Static Change of Log-Normal Distribution

Next, we consider a mid-scale range governed not by Gibrat’s law, (6), but by the non-Gibrat’s property in Equations (7) and (8). Since there is no Gibrat’s law, (6), Equation (11) cannot be transformed from a second expression to a third one. Using Equation (5), Equations (7) and (8) yield:

(17) $$\begin{array}{ccc} & & Q\left(R|x_{T}\right)=d\;R^{\;-1-t_{+}\left(x_{i}\right)+u_{+}\left(x_{i}\right)\ln R}\;\;\;\mathrm{for}\;\;\;R>1\;, \end{array}$$

(18) $$\begin{array}{ccc} & & Q\left(R|x_{T}\right)=d\;R^{\;-1+t_{-}\left(x_{i}\right)+u_{-}\left(x_{i}\right)\ln R}\;\;\;\mathrm{for}\;\;\;R<1\;. \end{array}$$

When R>1, Equations (17) and (18) rewrite the second expression of Equation (11):

(19) $$\begin{array}{c} \frac{P(x_{i})}{P(R^{1/\theta }x_{i})}=R^{\;1/\theta +t_{+}\left(x_{i}\right)-t_{-}\left(R^{1/\theta }x_{i}\right)-\left\{u_{+}\left(x_{i}\right)-u_{-}\left(R^{1/\theta }x_{i}\right)\right\}\ln R}\;. \end{array}$$

Expand this equation as R=1+ϵ(ϵ<<1). In Equation (19), $\epsilon^{0}$ order term is trivial. $\epsilon^{1}$ order term in the expansion from Equation (19) yields

(20) $$\begin{array}{c} \left[1+\theta \{t_{+}\left(x_{i}\right)-t_{-}\left(x_{i}\right)\}\right]P\left(x_{i}\right)+x_{i}\frac{dP(x_{i})}{dx_{i}}=0\;. \end{array}$$

Using Equation (20) to rewrite $dP(x_{i})/dx$ into $P(x_{i})$, $\epsilon^{2}$ order term in the expansion from Equation (19) yields:

(21) $$\begin{array}{c} x_{i}\left\{\frac{dt_{+}\left(x_{i}\right)}{d_{i}x}+\frac{dt_{-}\left(x_{i}\right)}{dx_{i}}\right\}+2\theta \left\{u_{+}\left(x_{i}\right)-u_{-}\left(x_{i}\right)\right\}=0\;. \end{array}$$

If $u_{-}\left(x_{i}\right)$ is eliminated using Equation (21), the expansion $\epsilon^{3}$ order term in Equation (19) yields:

(22) $$\begin{array}{c} 2\frac{dt_{+}\left(x_{i}\right)}{dx_{i}}+\frac{dt_{-}\left(x_{i}\right)}{dx_{i}}+6\theta \frac{du_{+}\left(x_{i}\right)}{dx_{i}}+x_{i}\{2\frac{d^{2}t_{+}\left(x_{i}\right)}{d^{2}x_{i}}+\frac{d^{2}t_{-}\left(x_{i}\right)}{d^{2}x_{i}}\}=0\;. \end{array}$$

When $u_{+}\left(x_{i}\right)$ is erased using Equation (22), the expansion $\epsilon^{4}$ order term in Equation (19) yields:

(23) $$\begin{array}{ccc} & & \frac{dt_{+}\left(x_{i}\right)}{dx_{i}}+\frac{dt_{-}\left(x_{i}\right)}{dx_{i}}+3x_{i}\{\frac{d^{2}t_{+}\left(x_{i}\right)}{d^{2}x_{i}}+\frac{d^{2}t_{-}\left(x_{i}\right)}{d^{2}x_{i}}\}\\ & & \;\;\;\;\;\;\;\;\;\;\;\;\;\;\;\;\;\;\;\;\;\;\;\;\;\;\;\;\;\;\;\;\;\;\;\;\;\;\;\;+{x_{i}}^{2}\{\frac{d^{3}t_{+}\left(x_{i}\right)}{d^{3}x_{i}}+\frac{d^{3}t_{-}\left(x_{i}\right)}{d^{3}x_{i}}\}=0\;. \end{array}$$

Solving this differential equation yields:

(24) $$\begin{array}{c} t_{+}\left(x_{i}\right)+t_{-}\left(x_{i}\right)=\frac{D_{+2}}{2}\ln^{2}\frac{x_{i}}{x_{0}}+D_{+1}\ln\frac{x_{i}}{x_{0}}+D_{+0}. \end{array}$$

Here, $D_{+2}$, $D_{+1}$, and $D_{+0}$ are integral constants. $x_{0}$ is a parameter introduced to smoothly connect the solution to the power-law distribution at $x=x_{0}$. If $t_{-}\left(x_{i}\right)$ is erased using Equation (24), the expansion $\epsilon^{5}$ order term in Equation (19) yields:

(25) $$\begin{array}{c} \frac{dt_{+}\left(x_{i}\right)}{dx_{i}}+7x_{i}\frac{d^{2}t_{+}\left(x_{i}\right)}{d^{2}x_{i}}+6{x_{i}}^{2}\frac{d^{3}t_{+}\left(x_{i}\right)}{d^{3}x_{i}}+{x_{i}}^{3}\frac{d^{4}t_{+}\left(x_{i}\right)}{d^{4}x_{i}}=0\;. \end{array}$$

The differential equation yields $t_{+}\left(x_{i}\right)$:

(26) $$\begin{array}{c} t_{+}\left(x_{i}\right)=\frac{D_{-3}}{3}\ln^{3}\frac{x_{i}}{x_{0}}+\frac{D_{-2}}{2}\ln^{2}\frac{x_{i}}{x_{0}}+2\alpha \ln\frac{x_{i}}{x_{0}}+D_{1}\;, \end{array}$$

where $D_{-3}$, $D_{-2}$, α, and $D_{1}$ are integral constants. Equations (26), (24), (22), (21), and (20) determine $t_{-}\left(x_{i}\right)$, $u_{\pm }\left(x_{i}\right)$, and $P(x_{i})$ as follows:

(27) $$\begin{array}{ccc} & & t_{-}\left(x_{i}\right)=-\frac{D_{-3}}{3}\ln^{3}\frac{x_{i}}{x_{0}}+\frac{D_{+2}-D_{-2}}{2}\ln^{2}\frac{x_{i}}{x_{0}}+\left(D_{+1}-2\alpha \right)\ln\frac{x_{i}}{x_{0}}+D_{2}\;, \end{array}$$

(28) $$\begin{array}{ccc} & & u_{+}\left(x_{i}\right)=-\frac{D_{-3}}{6\theta }\ln^{2}\frac{x_{i}}{x_{0}}-\frac{D_{+2}+D_{-2}}{6\theta }\ln\frac{x_{i}}{x_{0}}+D_{3}\;, \end{array}$$

(29) $$\begin{array}{ccc} & & u_{-}\left(x_{i}\right)=-\frac{D_{-3}}{6\theta }\ln^{2}\frac{x_{i}}{x_{0}}+\frac{2D_{+2}-D_{-2}}{6\theta }\ln\frac{x_{i}}{x_{0}}+\frac{D_{+1}}{2\theta }+D_{3}\;,\\ & & P\left(x_{i}\right)\propto {x_{i}}^{-\mu_{i}-1}\exp[-\frac{\theta D_{-3}}{6}\ln^{4}\frac{x_{i}}{x_{0}} \end{array}$$

(30) $$\begin{array}{ccc} & & \;\;\;\;\;\;\;\;\;\;\;\;\;\;\;\;\;\;\;\;\;\;\;\;\;\;\;\;+\frac{\theta (D_{+2}-2D_{-2})}{6}\ln^{3}\frac{x_{i}}{x_{0}}-\frac{\theta (4\alpha -D_{+1})}{2}\ln^{2}\frac{x_{i}}{x_{0}}]\;, \end{array}$$

where $D_{3}$ is an integral constant. In addition, we set $D_{2}=D_{+0}-D_{1}$, $\mu_{i}=\theta \left(D_{1}-D_{2}\right)$. The above is the result for R>1, and an identical result was obtained for R<1. They are obtained from the necessary conditions in the vicinity of R=1 in Equation (19). When we require $\mu_{i}=\theta \left(D_{1}-D_{2}\right)$, these solutions are sufficient, even not in the vicinity of R=1 of Equation (19). When the form of the growth-rate distribution is concretely assumed to be Equations (7) and (8), $\mu_{i}=\theta \left(D_{1}-D_{2}\right)$ becomes the reflection law.

The purpose of this paper is to confirm these analytical results with empirical data. Therefore, to the extent that as little generality as possible is lost, the simplest form is to assume that the $x_{i}$ dependency of $t_{\pm }\left(x_{i}\right)$ can be approximated to the first order of $\ln\frac{x_{i}}{x_{0}}$ [29]. In this case, from Equations (26) and (27), $D_{-3}=D_{\pm 2}=0$, and Equations (26)–(30) are simplified:

(31) $$\begin{array}{ccc} & & t_{+}\left(x_{i}\right)=\alpha_{+}\ln\frac{x_{i}}{x_{0}}+D_{1}\;, \end{array}$$

(32) $$\begin{array}{ccc} & & t_{-}\left(x_{i}\right)=\alpha_{-}\ln\frac{x_{i}}{x_{0}}+D_{2}\;, \end{array}$$

(33) $$\begin{array}{ccc} & & u_{+}\left(x_{i}\right)=D_{3}\;, \end{array}$$

(34) $$\begin{array}{ccc} & & u_{-}\left(x_{i}\right)=\frac{\alpha_{+}+\alpha_{-}}{2\theta }+D_{3}\;, \end{array}$$

(35) $$\begin{array}{ccc} & & P\left(x_{i}\right)\propto {x_{i}}^{-\mu_{i}-1}\exp\left[-\alpha_{i}\ln^{2}\frac{x_{i}}{x_{0}}\right]\;. \end{array}$$

Equation (35) is the log-normal distribution (2) itself, which is assumed to be a distribution in the mid-scale range, and shows the ease of handling this approximation.

Here, $\alpha_{+}=2\alpha$, $\alpha_{-}=D_{+1}-2\alpha$, and

(36) $$\begin{array}{c} \alpha_{i}=\frac{\theta }{2}\left(\alpha_{+}-\alpha_{-}\right)\;. \end{array}$$

Equation (36) is an analytic relationship linking log-normal parameter $\alpha_{i}$ and non-Gibrat’s parameter $\alpha_{\pm }$ in a quasi-statically varying system. As described above, to the best of our knowledge, only the data presented in this paper can empirically validate Equation (36).

Next, we determine the quasi-static time evolution of log-normal distribution (35). As in the previous subsection, using $P\left(x_{i}\right)dx_{i}=P\left(x_{i+1}\right)dx_{i+1}$, we obtain:

(37) $$\begin{array}{c} P\left(x_{i+1}\right)=P\left(x_{i}\right)\frac{dx_{i}}{dx_{i+1}}\propto {x_{i+1}}^{-\mu_{i}/\theta -1}\;\exp\left[-\frac{\alpha_{i}}{\theta^{2}}\ln^{2}\frac{x_{i}}{a{x_{0}}^{\theta }}\right]\;. \end{array}$$

Here, $\mu_{i}/\theta =\mu_{i+1}$ from Equation (16), and, denoting α at i+1 by $\alpha_{i+1}$, we obtain the following expression:

(38) $$\begin{array}{c} \theta^{2}=\frac{\alpha_{i}}{\alpha_{i+1}}\;. \end{array}$$

Using Equation (3), this expression can also be written:

(39) $$\begin{array}{c} \theta =\frac{\sigma_{i+1}}{\sigma_{i}}\;. \end{array}$$

This geometrically shows that the ratio of mid-scale spread $\sigma_{i}$, $\sigma_{i+1}$ in *i*, i+1 corresponds to slope θ of the quasi-time-reversal symmetry axis.

## 3. Results

In this section, we confirm the relationships between quasi-time-reversal symmetry and a change in the power-law exponent, between quasi-time-reversal symmetry and a change in the logarithmic standard deviation, and between non-Gibrat’s property and log-normal distribution in a quasi-statically changing system with empirical data.

### 3.1. Consistency between Quasi-Time-Reversal Symmetry and Changes of Power-Law Index

**Figure 11:**
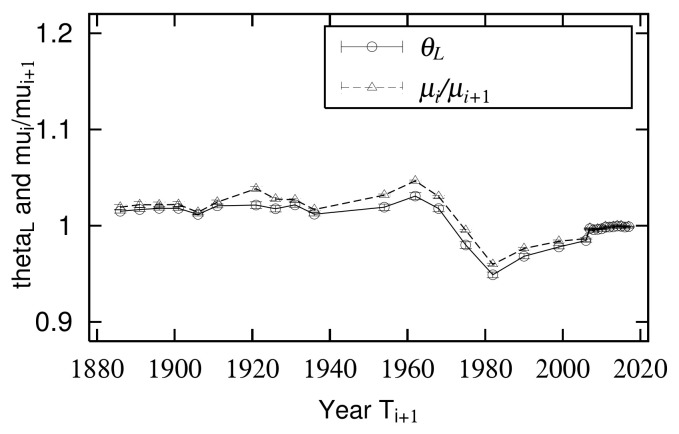
Comparison of time course of slope $\theta_{L}$ of symmetry axis of quasi-time-reversal symmetry in large-scale region and ratio of power-law index $\mu_{i}/\mu_{i+1}$.

The temporal variations of quasi-time-reversal symmetry parameters $\theta_{L}$ measured in the large-scale region are shown in Figure 6. From the discussion in Section 2.5, the relationship between $\theta_{L}$ and the time change of exponent μ is given by Equation (16). Figure 11 juxtaposes $\mu_{i}/\mu_{i+1}$ calculated from Figure 3 and $\theta_{L}$. Note that the years in which urban populations are measured are not evenly spaced (Table 1). This figure confirms that Equation (16) holds in the empirical data and supports the analytical discussion in Section 2.5.

### 3.2. Consistency of Quasi-Time-Reversal Symmetry and Changes in Logarithmic Standard Deviation

**Figure 12:**
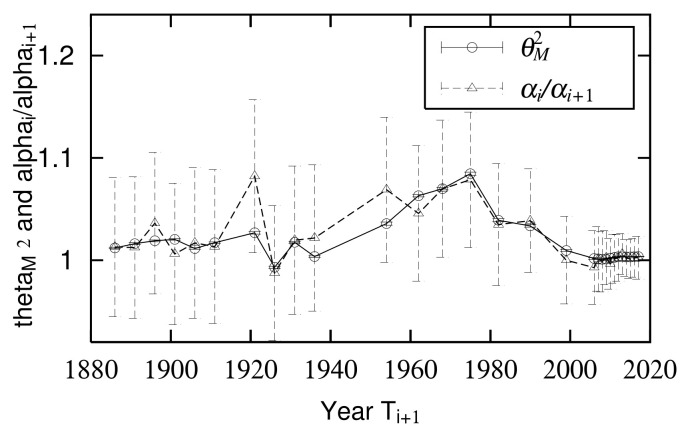
Comparison of time course of slope ${\theta_{M}}^{2}$ of symmetry axis of quasi-time-reversal symmetry in mid-scale region and ratio of α $\alpha_{i}/\alpha_{i+1}$.

The temporal variations of quasi-time-reversal symmetry parameters $\theta_{M}$ measured in the mid-scale region are shown in Figure 6. Based on the discussion in Section 2.6, the relationships between $\theta_{M}$ and the time variation of and logarithmic standard deviation σ or α are given by Equations (38) or (39). Figure 12 is a side-by-side drawing of $\alpha_{i}/\alpha_{i+1}$ calculated from Figure 3 and ${\theta_{M}}^{2}$. As shown in Figure 11, the measured values are not arranged at regular intervals because the intervals between years where urban population was measured are not constant (Table 1). This figure confirms that Equation (39) holds in the empirical data and supports the analytical discussion in Section 2.6.

### 3.3. Consistency between Non-Gibrat’s Property and Log-Normal Distribution

**Figure 13:**
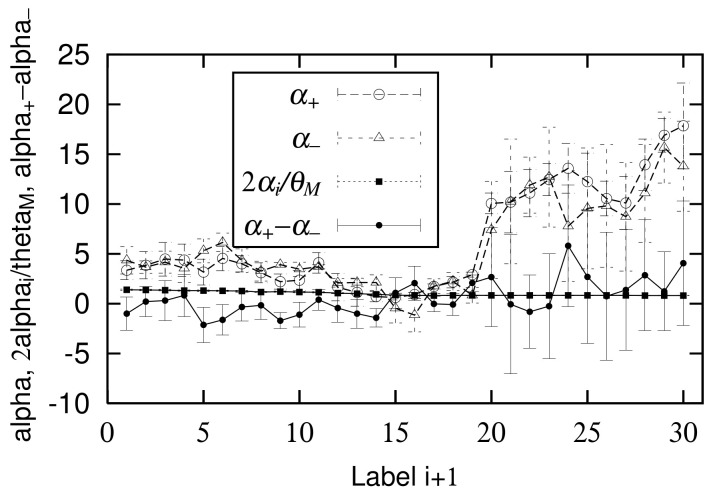
Comparison of time course of $\alpha_{\pm }$, $2\alpha_{i}/\theta_{M}$, and $\alpha_{+}-\alpha_{-}$.

Finally, in the empirical data, we confirm the relationship between logarithmic standard deviation $\alpha_{i}$ of the log-normal distribution and parameters $\alpha_{\pm }$ of the non-Gibrat’s property, and parameter $\theta_{M}$ of the quasi-time-reversal symmetry, shown analytically in Section 2.6. As noted in Section 2.6, the simplest approximation that retains as much generality as possible is $t_{\pm }\left(x_{i}\right)$, which becomes a linear function of $\ln x_{i}$, such as Equations (31) and (32), whose coefficients are $\alpha_{\pm }$. *c*, $u_{\pm }$ are constants that are independent of $x_{i}$, including Equations (7), (8), (33), and (34). Figure 9 and Figure 10 in Section 2.4 show the dependency of $t_{\pm }$, $u_{\pm }$, and *c* on $\log_{10}x_{i}$ for 1962 to 1968 (i=13) and 2015 to 2016 (i=28). In these figures, we first confirmed that *c* has no $x_{i}$ dependency, as described in Section 2.6. In the range of $10^{1}<x_{i}<10^{3.5}$ at least, we also confirmed the approximation that $u_{\pm }$ also has no $x_{i}$ dependency. The regression analyses on range $10^{1}<x_{i}<10^{3.5}$ yield $\alpha_{+}=0.71\pm 0.52$, $\alpha_{-}=2.12\pm 0.78$ for Figure 9 and $\alpha_{+}=16.9\pm 1.7$, $\alpha_{-}=15.7\pm 3.6$ for Figure 10. Similarly, applying a log-normal distribution (2) or (35) to Figure 1 and Figure 2 yields parameters $\alpha_{13}=0.96\pm 0.04$ and $\alpha_{28}=0.82\pm 0.01$. In Figure 4 and Figure 5, the mid-scale quasi-time-reversal symmetry parameters are estimated as $\theta_{M}=1.03\pm 0.00$ and $\theta_{M}=1.00\pm 0.00$. The time variation over the entire period of each parameter is shown in Figure 13.

The horizontal axis of Figure 13 is not a year but a label (i+1) that simplifies observing the points after 2006 and 2007 (i+1=20) where the data interval is 1 year. In this figure, $2\alpha_{i}/\theta_{M}$ continues to decrease until around 1968 (i+1=14), and thereafter it remains almost constant. However, in Figure 13, the change is inconspicuous and the error is small because the scale on the vertical axis is large. By comparison, $\alpha_{\pm }$ changes significantly. The values after 2007 (i+1=20), where the observation years are separated by one year, are especially different from the values before 2007. However, difference $\alpha_{+}-\alpha_{-}$ in any year is small and often within the error of $2\alpha_{i}/\theta_{M}$. These observations confirmed the analytical conclusion of Section 2.6, Equation (36), in the empirical data.

## 4. Discussion

We analytically derived the following three relations among the quasi-time-reversal symmetry, Gibrat’s law, and the non-Gibrat property, all of which were observed in the urban population data of France, and confirmed them in the empirical data. The first is the relation between the time variation of the power law and the quasi-time-reversal symmetry in large-scale range $\left(\mu_{i}/\mu_{i+1}=\theta_{L}\right)$. The second is the relation between the time variation of the log-normal distribution between the quasi-time-reversal symmetry in the mid-scale range $\left(\alpha_{i}/\alpha_{i+1}={\theta_{M}}^{2}\right)$. The third is the relation among the parameters of the non-Gibrat’s property, the log-normal distribution, and the quasi-time-reversal symmetry $\left(\alpha_{+}-\alpha_{-}=2\alpha_{i}/\theta \right)$.

This paper addressed the non-Gibrat’s property of the growth-rate distribution with a convex downward curvature on both logarithmic axes. A previous study on published land prices assumed that the growth-rate distribution was linear on both logarithmic axes. We struggled to directly observe the changes of the growth-rate distribution in the mid-scale range, because of a data shortage in one period. However, using France’s urban population data, we directly observed them in a quasi-statically changing system and confirmed the consistency between the parameters of the non-Gibrat’s property and the log-normal distribution. This observation is the first research result on empirical data, to the best of our knowledge.

On the one hand, the evaluation of power-law exponent μ becomes the regression analysis of one parameter. On the other hand, since the evaluation of the non-Gibrat’s property becomes the estimation of three parameters, $c,t_{\pm }$, and $u_{\pm }$, the error must be larger than the exponent. Therefore, it was difficult to observe temporal changes in the relationship between the non-Gibrat’s property and the log-normal distribution. However, we confirmed that the non-Gibrat’s parameter $\left(\alpha_{\pm }\right)$ increased as the interval of the measurement period decreased and that the difference $\left(\alpha_{+}-\alpha_{-}\right)$ coincided with the parameter $\left(2\alpha_{i}/\theta_{M}\right)$ of the log-normal distribution and the quasi-time reversal symmetry predicted by the analytical argument at many measurement points within the error range. This research result is important because, even when the non-Gibrat’s form is more complicated, we can confirm the consistency between analytical discussion and empirical data.

Finally, we discuss how this paper can be viewed from the perspective of urban spatial networks. According to the urban population distribution in France, power-law index μ gradually decreased from 1876 to 1968, indicating that the disparity among cities in the large-scale range widened during this period. This means that France’s population became concentrated in large cities. After 1968, power-law index μ started to increase, suggesting that the population concentration in large-scale cities was gradually being diffused. Concentrated populations in large-scale cities must move to mid-scale cities, as evidenced by the acceleration of mid-scale city spread σ since 1968. In this way, we can understand the micro-scale phenomenon as the population moving from large-scale to mid-scale cites based on the change of macroscopic distributions. When constructing a microscopic model of population movement between cities, a model must be designed in such a way that it is consistent with this macroscopic nature, which will shape its construction.

## Figures and Tables

**Table 1:** Number of communes and their total population in France between 1876 and 2017, as listed in database: Observation data are assigned serial numbers *i* from oldest to newest.

Mark (i)	Year $\left(T_{i}\right)$	Communes	Population
0	1876	34,500	38,173,561
1	1881	34,500	38,969,724
2	1886	34,500	39,508,491
3	1891	34,500	39,660,067
4	1896	34,500	39,871,028
5	1901	34,500	40,390,113
6	1906	34,500	40,780,507
7	1911	34,500	41,147,539
8	1921	34,500	38,932,989
9	1926	34,500	40,458,773
10	1931	34,500	41,541,494
11	1936	34,860	41,813,397
12	1954	34,946	43,394,688
13	1962	34,972	47,376,787
14	1968	34,972	50,798,112
15	1975	34,972	53,764,064
16	1982	34,972	55,569,542
17	1990	34,972	58,040,659
18	1999	34,972	60,149,901
19	2006	34,972	63,186,117
20	2007	34,972	63,600,690
21	2008	34,972	63,961,859
22	2009	34,972	64,304,500
23	2010	34,972	64,612,939
24	2011	34,972	64,933,400
25	2012	34,972	65,241,241
26	2013	34,972	65,564,756
27	2014	34,972	65,907,160
28	2015	34,972	66,190,280
29	2016	34,972	66,361,658
30	2017	34,972	66,524,339

## Data Availability

Restrictions apply to the availability of these data. Data were obtained from: Chiffres detailles—Series historiques de population (1876 a 2017)” and are available with the permission of the National Institute of Statistics and Economic Studies at: https://www.insee.fr/en/accueil (accessed on January 2020).
